# A nomogram clinical model for predicting preoperative lower limb venous thrombosis in knee ligament injuries: a retrospective study

**DOI:** 10.3389/fmed.2025.1486625

**Published:** 2025-03-28

**Authors:** Jincai Duan, Tianjie Xiao, Tianyou Xing, Wei Qin, Zhihui Wang, Haoduan Dou, Di Wu, Yuanliang Du

**Affiliations:** ^1^Department of Joint Surgery, Affiliated Hospital of Chengde Medical College, Hebei, China; ^2^Graduate School of Chengde Medical College, Hebei, China; ^3^Department of Traditional Chinese Medicine, Affiliated Hospital of Chengde Medical College, Hebei, China; ^4^Department of Minimally Invasive Spine Surgery, Affiliated Hospital of Chengde Medical College, Hebei, China

**Keywords:** knee ligament injuries, lower limb venous thrombosis, nomogram, deep vein thrombosis, predictive model

## Abstract

**Purpose:**

To identify independent risk factors for preoperative lower limb venous thrombosis (LLVT) in knee ligament injuries and to develop a diagnostic prediction model based on these factors.

**Methods:**

Patients with knee ligament injuries who presented to our hospital between July 2021 and December 2023 were included in this study. Logistic regression analysis was utilized to determine independent risk factors for preoperative LLVT in knee ligament injuries and to construct a diagnostic prediction model. The diagnostic performance of the model was evaluated using receiver operating characteristic curves (ROC) and calibration curves.

**Results:**

Compared with the None-LLVT group, the LLVT group showed statistically significant differences in age, gender, damaged ligament site, injury-examination time, low density lipoprotein (LDL), glucose (G), D-dimer, and fibrinogen degradation products (FDP) (*P* < 0.05). Multivariate logistic regression analysis showed that gender (*P* = 0.006, 95% CI [1.647-19.450]), damaged ligament site (*P* = 0.016, 95% CI [1.385-23.060]), and D-dimer > 0.55 mg/L (*P* < 0.001, 95% CI [3.029-37.845]) were independent risk factors for preoperative LLVT in patients with knee ligament invasion. The ROC showed good diagnostic efficacy, with an area under the curve (AUC) of 0.888, and the calibration curves showed good agreement (mean absolute error = 0.013).

**Conclusion:**

Gender, damaged ligament site, and D-dimer level can be used as independent risk factors for the preoperative prediction of LLVT, and the nomogram model proposed in this study can better assist clinicians in making clinical decisions.

## 1 Introduction

In recent years, with the popularity of the concept of national sports, an increasing number of people participate in sports, followed by an increase in the number of injuries associated with sports, especially in the process of non-contact sports. Cruciate ligament injury have become a more common knee joint sports injury, among which the anterior cruciate ligament (ACL) injuries are more common (1). A study of ACL injury rates in college sports showed that 59% of ACL injuries in men were contact injuries and 60% of ACL injuries in women were non-contact injuries (2). Posterior cruciate ligament (PCL) injuries are usually associated with other ligament, menisci, and cartilage injuries (3). Inadequate treatment of cruciate ligament injuries can have a serious impact on the knee function and quality of life. It has been shown (4, 5) that the short-term outcome of ACL rupture is an inability to participate in activities and a long functional rehabilitation time, and that in terms of long-term outcomes, ACL reconstruction with or without ACL reconstruction increases the patient’s risk of developing osteoarthritis and is often accompanied by meniscus damage.

Surgery is an effective treatment option for patients with knee injuries. It has been suggested that the first-line treatment for patients with ACL rupture is ACL reconstruction followed by rehabilitation (6). Santiago et al. (3) stated that surgical treatment of symptomatic simple and combined PCL injuries is recommended to restore joint stability and improve knee function. Surgical treatment of simple PCL also reduces the incidence of secondary osteoarthritis (7).

Lower limb venous thrombosis (LLVT) is the formation of new blood clots in the veins of the lower limbs, which can be severe enough to lead to the development of pulmonary embolism (PE) and death. When the key components of Virchow’s triad, namely slow blood flow, endothelial damage, and hypercoagulability, are met simultaneously, the chances of thrombosis are significantly elevated. More and more people are opting for arthroscopic surgery to treat cruciate ligament injuries for early recovery of knee function, improvement of symptoms, and quality of life; however, the potential for deep vein thrombosis and its sequelae are also present. A Meta-analysis noted that the incidence of DVT after knee arthroscopy was 3.1-17.9% (8). It has been shown (9) that post-thrombotic syndrome without intervention gradually increases from 23% at 2 years to more than 49% at 5-10 years, and may lead to amputation in the worst cases. However, controversy remains regarding whether prophylactic anticoagulation should be administered. Yeo et al. (10) suggested that there is a significant increase in the incidence of DVT in the perioperative period of knee arthroscopy and that prophylactic anticoagulation is necessary in some patients. It has been suggested that patients with no risk factors for thrombosis, not at high risk for thrombosis, less than 45 years of age, and early functional exercise do not need anticoagulant therapy for thromboprophylaxis (11–13).

It is now clinically observed that venous thrombosis occurs after a knee injury and before surgical treatment and that the probability of LLVT increases considerably after surgical treatment. Song et al. (14) noted that patients with a preoperative diagnosis of LLVT had a 66.7% probability of developing thrombus at the same site postoperatively. Thus, the risk of venous thrombosis is unpredictable until symptoms develop, and both patients and clinicians may overlook this danger. This study aimed to investigate the risk factors for preoperative LLVT in patients with knee ligament injuries and to provide clinicians and patients with a portable predictive model to assist in the diagnosis of LLVT in order to adopt a personalized treatment plan.

## 2 Materials and methods

### 2.1 Data source and cohort selection

**FIGURE 1:**
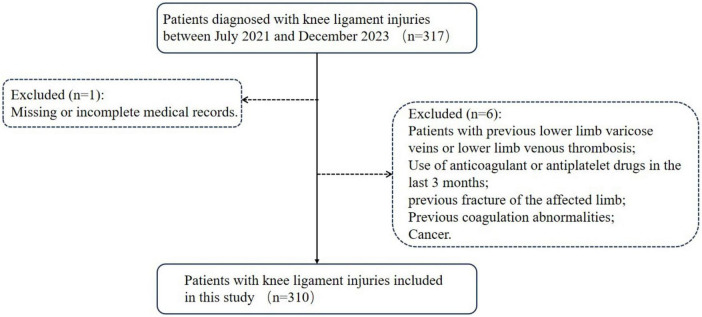
Flowchart for inclusion and exclusion of preoperative lower limb venous thrombosis (LLVT) in knee ligament injuries.

Through the hospital’s electronic medical record system, 317 patients with a diagnosis of knee ligament injury who visited our hospital between July 2021 and December 2023 were included. The inclusion and exclusion process for this study is presented in Figure 1. Patients who met one or more of the following conditions were excluded from this study: patients with missing or incomplete medical records, previous lower limb varicose veins or lower limb venous thrombosis, use of anticoagulant or antiplatelet drugs in the last 3 months, previous fracture of the affected limb, Previous coagulation abnormalities, and cancer.

Patient information included in the study included: Age, Gender (Male or Female), Blood type (A, B, AB, and O), Body mass index (BMI) (< 18.5 kg/m^2^, 18.5-23.9 kg/m^2^, 24-27.9 kg/m^2^, ≥ 28 kg/m^2^), History of smoking and alcohol consumption, Comorbid history (including hypertension, diabetes, coronary heart disease, cerebral infarction, others, and none), Damaged ligament site (ACL, PCL, ACL+PCL, ACL/PCL+medial/lateral collateral ligament [MCL/LCL]), Lateral injury, Injury-examination time, Triglyceride (TG), High density lipoprotein (HDL), Low density lipoprotein (LDL), Glucose (G), Plasma fibrinogen (FIB), D-dimer, Fibrinogen degradation product (FDP), Blood sedimentation rate (ESR), and the results of preoperative ultrasonography of LLVT (without venous thrombosis or venous hemorrhage of the lower limb).

All patients underwent ultrasonography and were then co-diagnosed by two experienced ultrasonographers. In case of disputed diagnosis, the decision was made in consultation with the supervising physician. DVT and intermuscular vein thrombosis (IVT) were included in this study.

### 2.2 Statistical analysis

All data were analyzed using SPSS 25.0 (IBM Corporation, Armonk, NY, United States). Continuous variables are expressed as mean ± standard deviation, and categorical variables are expressed as numbers and percentages. All factors are normally distributed. Depending on the type of data, independent sample *t*-tests or Mann-Whitney tests were used for comparisons between continuous and Pearson chi-square, and rank-sum tests were used for categorical variables to assess between-group differences in patients with and without LLVT. Univariate and multivariate logistic regression analyses were performed on the included indicators to determine the risk factors of preoperative LLVT in patients with knee ligament injuries. A nomogram clinical prediction model was developed using R 4.3.2 software, based on the “rms” R package using multivariate logistic analysis. The diagnostic predictive value of the model for patients with knee ligament injuries who developed preoperative LLVT was assessed using the receiver operating characteristic (ROC) Curve. Calibration curves were used to validate the predictive performance of the nomogram. *P* < 0.05 were considered statistically significant differences.

### 2.3 Ethical considerations

As this was a retrospective study, and data were analyzed anonymously, informed consent was therefore waived by the committee. This study was approved by the Ethical Review Committee of Chengde Medical College Hospital. This study was conducted in accordance with the guiding principles of the 1964 Declaration of Helsinki and its subsequent amendments.

## 3 Results

### 3.1 Patient selection and characteristics

The flow chart for the inclusion and exclusion of patients is shown in Figure 1. From July 2021 to December 2023, 317 patients with knee ligament injuries were enrolled, 7 ineligible patients were excluded according to the exclusion criteria, and 310 patients were included in the study; 283 patients (91.3%) without preoperative LLVT and 27 patients (8.7%) with preoperative LLVT were included in the study. Patients were categorized into the None-LLVT and LLVT groups based on the presence or absence of preoperative LLVT. In the LLVT group, there were 3 cases of DVT and 24 had IVT.

The youngest patient in this study was 13 years old, and the oldest patient was 68 years old, with a mean age of 41.45 ± 13.58 years. The basic information of the included patients is presented in Table 1. There were 166 men and 144 women, the prevalence of preoperative LLVT was significantly higher in women than in men (6.1% of all patients and 13.2% of the incidence population). Among the patients, 177 had left knee injuries, 130 had right knee injuries, and 3 had bilateral knee injuries. Grouped according to the acute and chronic phase of knee injury (≤21 days and >21 days), there were 206 patients (66.5%) with acute knee ligament injuries and 104 patients (33.5%) with chronic knee ligament injuries.

**TABLE 1:** Demographic and clinical characteristics of patients with knee ligament injuries with and without lower limb venous thrombosis (LLVT).

	Total (*N* = 310)		
	**None-LLVT group** **(*n* = 283)**	**LLVT group** **(*n* = 27)**	**X^2^/t, P**
**Age** (years)	40.76 ± 13.71	48.67 ± 9.67	2.925, 0.004
**Gender**			6.803, 0.009
Male	158 (95.2%)	8 (4.8%)	
Female	125 (86.8%)	19 (13.2%)	
**Blood type**			2.727, 0.436
A	73 (93.6%)	5 (6.4%)	
B	98 (89.1%)	12 (10.9%)	
AB	25 (86.2%)	4 (13.8%)	
O	87 (93.5%)	6 (6.5%)	
BMI (kg/m^2^)			2.131, 0.546
<18.5	5 (100.0%)	0 (0.0%)	
18.5∼23.9	75 (88.2%)	10 (11.8%)	
24∼27.9	127 (91.4%)	12 (8.6%)	
≥ 28	76 (93.8%)	5 (6.2%)	
**Smoking**			1.255, 0.263
Yes	81 (94.2%)	5 (5.8%)	
None	202 (90.2%)	22 (9.8%)	
**Drinking**			1.131, 0.288
Yes	91 (93.8%)	6 (6.2%)	
None	192 (90.1%)	21 (9.9%)	
**Comorbid history**			10.726, 0.057
Hypertension	23 (85.2%)	4 (14.8%)	
Diabetes	6 (75.0%)	2 (25.0%)	
Coronary heart disease	1 (100.0%)	0 (0.0%)	
Cerebral infarction	25 (83.3%)	5 (16.7%)	
Others	7 (77.58%)	2 (22.2%)	
None	221 (94.0%)	14 (6.0%)	
**Damaged ligament**			14.653, 0.002
ACL	157 (96.3%)	6 (3.7%)	
PCL	18 (85.7%)	3 (14.3%)	
ACL+PCL	13 (100.0%)	0 (0.0%)	
ACL/PCL+MCL/LCL	95 (84.1%)	18 (15.9%)	
**Lateral**			5.008, 0.082
Left	158 (89.3%)	19 (10.7%)	
Right	123 (94.6%)	7 (5.4%)	
Bilateral	2 (66.7%)	1 (33.3%)	
**Injury-examination time (days)**			4.656, 0.031
≤ 21	183 (88.8%)	23 (11.2%)	
>21	100 (96.2%)	4 (3.8%)	
TG (mmol/L)	1.80 ± 1.23	1.64 ± 0.92	0.626, 0.532
HDL (mmol/L)	1.15 ± 0.25	1.21 ± 0.22	–1.047, 0.296
LDL (mmol/L)	2.89 ± 0.65	3.21 ± 0.86	–2.216, 0.027
G (mmol/L)	5.45 ± 1.93	6.48 ± 2.94	–2.411, 0.017
FIB (g/L)	2.83 ± 0.78	3.01 ± 0.55	–1.179, 0.239
D-dimer (mg/L)	0.28 ± 0.29	1.80 ± 1.71	–13.277, <0.001
FDP (μg/L)	2.69 ± 0.78	5.82 ± 5.74	–8.481, <0.001
ESR (mm/1 h)	12.16 ± 11.33	12.35 ± 7.31	–0.076, 0.940

LLVT, lower limb venous thrombosis; BMI, body mass index; ACL, anterior cruciate ligament; PCL, posterior cruciate ligament; MCL, medial collateral ligament; LCL, lateral collateral ligament; TG, triglyceride; HDL, high density lipoprotein; LDL, low density lipoprotein; G, glucose; FIB, plasma fibrinogen; FDP, fibrinogen degradation products; ESR, blood sedimentation rate.

### 3.2 Between-group comparison of none-LLVT vs. LLVT

**FIGURE 2:**
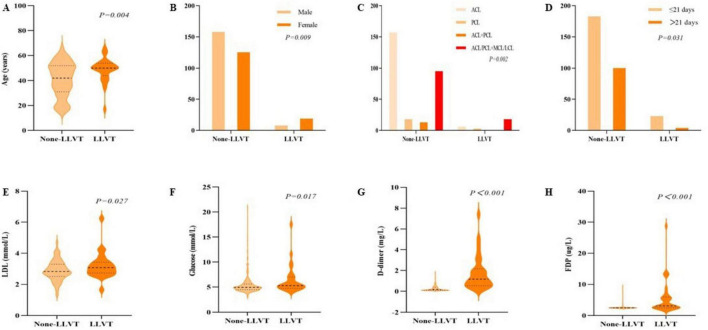
Between-group comparisons between the none-lower extremity venous thrombosis (None-LLVT) group and the lower extremity venous thrombosis (LLVT) group. Variables included were **(A)** age, **(B)** gender, **(C)** site of ligament damage, and **(D)** injury-examination time, **(E)**. low-density lipoprotein (LDL), **(F)** glucose, **(G)** D-dimer, and **(H)** fibrinogen degradation products (FDP).

The demographic and clinical characteristics of patients with knee ligament injuries with and without LLVT are summarized in Table 1. In this study, the mean age was 40.76 ± 13.71 years in the None-LLVT group and 48.67 ± 9.67 years in the LLVT group, with a significant difference between the groups (*P* = 0.004). There were 8 males (4.8%) and 19 females (13.2%) in the LLVT group, the incidence of which was significantly higher in females than in males, with a statistically significant difference between the groups (*P* = 0.009). Among the included patients, 163 (52.6%) had ACL injuries, 21 (6.8%) had PCL injuries, 13 (4.2%) had ACL+PCL injuries, and 113 (36.4%) had ACL/PCL+MCL/LCL injuries with a significant difference between the two groups (*P* = 0.002). In the LLVT group, 23 (11.2%) patients belonged to the acute phase and 4 (3.8%) patients were in the chronic phase, with a statistically significant difference between the two groups (*P* = 0.031). In laboratory tests, LDL, G, D-dimer, and FDP levels were statistically different between the groups (*P* = 0.027, *P* = 0.017, *P* < 0.001, and *P* < 0.001, respectively). Statistically significant differences between the two groups are shown in Figure 2.

### 3.3 Analysis of risk factors for preoperative LLVT

The results of the univariate logistic regression analysis for the occurrence of preoperative LLVT in patients with knee ligament injuries are summarized in Table 2. In the univariate analysis, the risk factors for patients to develop preoperative LLVT were age (*P* = 0.005, 95% CI [1.015-1.090]); females were more likely to develop thrombosis compared with males (*P* = 0.015, 95% CI [1.272-7.085]); and patients with comorbid cerebral infarctions were more likely to develop LLVT compared with patients with no co-morbid history (*P* = 0.041, 95% CI [1.049-9.501]); PCL injury and ACL/PCL+MCL/LCL injury increased the risk of LLVT compared with ACL injury (*P = 0.049*, 95% CI [1.004-18.953]; *P* = 0.001, 95% CI [1.901-12.928], respectively); Patients with knee ligament injuries in the chronic phase had a lower risk of preoperative LLVT compared with those in the acute phase (*P* = 0.039, 95% CI [0.107-0.946]; higher levels of LDL, G, and FDP also increased the prevalence of preoperative LLVT (*P* = 0.030, 95% CI [1.064-3.365]; *P* = 0.028, 95% CI [1.017-1.348]; *P* < 0.001, 95% CI [1.425-2.445], respectively), and patients with > 0.55 mg/L had a higher risk of LLVT compared with patients with normal D-dimer levels (*P* < 0.001, 95% CI [7.542-46.016]).

**TABLE 2:** Univariate logistic regression analysis of factors associated with lower limb venous thrombosis (LLVT) in patients with knee ligament injuries.

Subject characteristics	Total (*N* = 310)			
	**Coefficient**	**Std. Error**	***P*-value**	**95% CI**
**Age**	0.051	0.018	0.005	1.052 (1.015-1.090)
**Gender**				
Male	–	–	1.000	1 (reference)
Female	1.099	0.438	0.012	3.002 (1.272-7.085)
**Blood type**				
A	–	–	1.000	1 (reference)
B	0.581	0.554	0.295	1.788 (0.603-5.298)
AB	0.848	0.710	0.232	2.336 (0.581-9.388)
O	0.007	0626	0.991	1.007 (0.295-3.434)
**BMI (kg/m^2^)**				
18.5∼23.9	–	–	1.000	1 (reference)
<18.5	–	–	NA	NA
24∼27.9	–0.344	0.452	0.446	0.709 (0.292-1.720)
≥ 28	–0.706	0.571	0.216	0.493 (0.161-1.512)
**Smoking**				
Yes	–	–	1.000	1 (reference)
None	0.568	0.513	0.268	1.764 (0.646-4.818)
**Drinking**				
Yes	–	–	1.000	1 (reference)
None	0.506	0.480	0.292	1.659 (0.647-4.251)
**Co-morbid history**				
None	–	–	1.000	1 (reference)
Hypertension	1.010	0.608	0.097	2.745 (0.834-9.036)
Diabetes	1.660	0.862	0.054	5.262 (0.972-28.488)
Coronary heart disease	–	–	NA	NA
Cerebral infarction	1.150	0.562	0.041	3.157 (1.049-9.501)
Others	1.506	0.848	0.076	4.510 (0.856-23.761)
**Damaged ligament**				
ACL	–	–	1.000	1 (reference)
PCL	1.473	0.750	0.049	4.361 (1.004-18.953)
ACL+PCL	–	–	NA	NA
ACL/PCL+MCL/LCL	1.601	0.489	0.001	4.958 (1.901-12.928)
**Lateral**				
Left	–	–	1.000	1 (reference)
Right	–0.748	0.458	0.103	0.473 (0.193-1.162)
Bilateral	1.425	1.249	0.254	4.158 (0.360-48.047)
**Injury-examination time (days)**				
≤ 21	–	–	1.000	1 (reference)
>21	–1.145	0.556	0.039	0.318 (0.107-0.946)
**TG (mmol/L)**	–0.129	0.205	0.531	0.879 (0.588-1.315)
**HDL (mmol/L)**	0.869	0.831	0.296	2.384 (0.468-12.155)
**LDL (mmol/L)**	0.638	0.294	0.030	1.892 (1.064-3.365)
**G (mmol/L)**	0.158	0.072	0.028	1.171 (1.017-1.348)
**FIB (g/L)**	0.304	0.258	0.239	1.355 (0.817-2.248)
**D-dimer (mg/L)**				
0-0.55	–	–	1.000	1 (reference)
>0.55	2.925	0.461	<0.001	18.629 (7.542-46.016)
**FDP (μg/L)**	0.624	0.138	<0.001	1.866 (1.425-2.445)
**ESR (mm/1 h)**	0.002	0.021	0.939	1.002 (0.962-1.043)

LLVT, lower limb venous thrombosis; BMI, body mass index; ACL, anterior cruciate ligament; PCL, posterior cruciate ligament; MCL, medial collateral ligament; LCL, lateral collateral ligament; TG, triglyceride; HDL, high density lipoprotein; LDL, low density lipoprotein; G, glucose; FIB, plasma fibrinogen; FDP, fibrinogen degradation products; ESR, blood sedimentation rate.

Based on the results of the univariate logistic regression analysis, statistically significant factors were included in the multivariate logistic regression analysis; the results are summarized in Table 3. The study showed that gender, damaged ligament site, and D-dimer>0.55 mg/L were independent risk factors for the occurrence of preoperative LLVT in knee ligament injuries (*P* = 0.006, 95% CI [1.647-19.450]; *P* = 0.016, 95% CI [1.385-23.060]; and *P* < 0.001, 95% CI [3.029-37.845], respectively).

**TABLE 3:** Multivariable logistic regression analysis of factors associated with lower limb venous thrombosis (LLVT) in patients with knee ligament injuries.

Subject characteristics	Total (N = 310)			
	**Coefficient**	**Std. Error**	***P*-value**	**95% CI**
**Age**	–0.020	0.026	0.427	0.980 (0.932-1.030)
**Gender**				
Male	–	–	1.000	1 (reference)
Female	1.734	0.630	0.006	5.661 (1.647-19.450)
**Co-morbid history**				
None	–	–	1.000	1 (reference)
Hypertension	0.476	0.851	0.576	1.609 (0.304-8.524)
Diabetes	1.035	1.607	0.519	2.816 (0.121-65.635)
Coronary heart disease	–	–	NA	NA
Cerebral infarction	0.974	0.771	0.207	2.647 (0.584-12.000)
Others	2.017	1.316	0.125	7.517 (0.570-99.209)
**Damaged ligament**				
ACL	–	–	1.000	1 (reference)
PCL	1.522	1.137	0.181	4.580 (0.493-42.515)
ACL+PCL	–	–	NA	NA
ACL/PCL+MCL/LCL	1.732	0.717	0.016	5.651 (1.385-23.060)
**Injury-examination time (days)**				
≤21	–	–	1.000	1 (reference)
>21	0.023	0.758	0.975	1.024 (0.232-4.524)
LDL (mmol/L)	0.699	0.426	0.101	2.012 (0.873-4.641)
G (mmol/L)	0.005	0.121	0.964	1.005 (0.793-1.274)
**D-dimer (mg/L)**				
0-0.55	–	–	1.000	1 (reference)
>0.55	2.371	0.644	<0.001	10.706 (3.029-37.845)
FDP (μg/L)	0.242	0.151	0.110	1.273 (0.946-1.713)

LLVT, lower limb venous thrombosis; ACL, anterior cruciate ligament; PCL, posterior cruciate ligament; MCL, medial collateral ligament; LCL, lateral collateral ligament; LDL, low density lipoprotein; G, glucose; FDP, fibrinogen degradation products.

### 3.4 Development and validation of personalized predictive model

**FIGURE 3:**
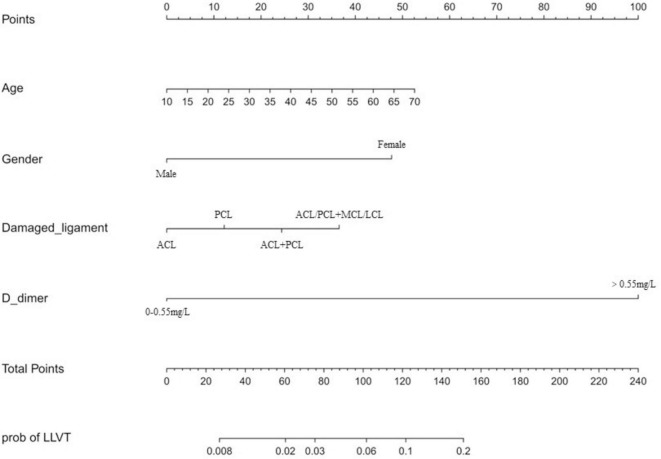
Diagnostic predictive nomogram for preoperative lower limb venous thrombosis (LLVT) in knee ligament injuries.

**FIGURE 4:**
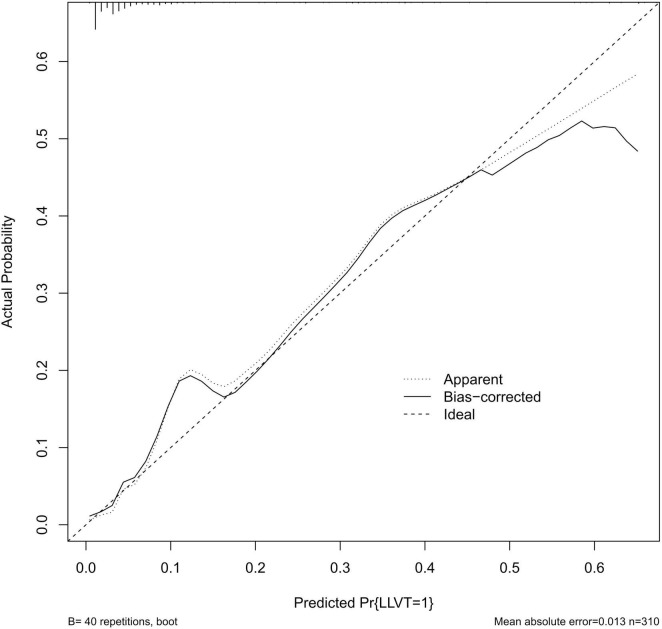
Receiver operating characteristic (ROC) curve plotted from logistic regression analysis.

**FIGURE 5:**
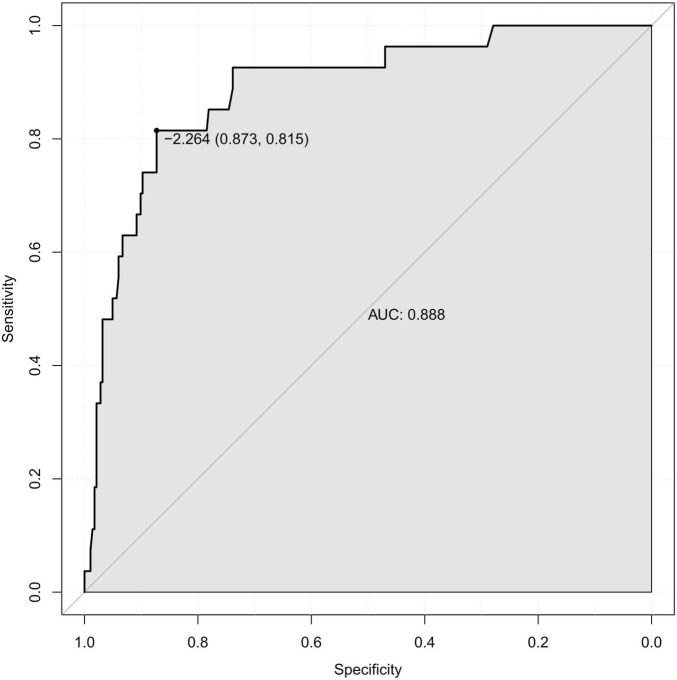
Calibration curves for prediction models.

Previous studies have shown that advanced age is a clear and important risk factor for venous thrombosis (10, 13). Combined with the results of the multivariate logistic regression analysis based on this study, age, gender, damaged ligament site, and D-dimer level were incorporated into the construction of the diagnostic prediction model, as shown in Figure 3. The model was evaluated using the ROC curve, reflecting the good predictive value of the model in this study, with an area under the curve (AUC) of 0.888 (specificity of 87.3% and sensitivity of 81.5%) (Figure 4). The calibration curve showed good agreement between the predicted value and actual risk, with an average absolute error of 0.013 (Figure 5). These results indicate that the diagnostic prediction model can better predict the incidence of preoperative LLVT in patients with knee ligament injuries and guide clinicians in making better decisions.

## 4 Discussion

Wakabayashi et al. (15) noted that the incidence of preoperative asymptomatic deep vein thrombosis was as high as 17.4% in patients admitted for knee osteoarthritis (KOA). In this study, the incidence of preoperative LLVT in patients admitted for knee ligament injuries was 8.7%, which is lower than the incidence in patients with KOA. However, it has been shown that in total hip arthroplasty (THA) patients with a preoperative diagnosis of LLVT, the probability of recurrent thrombosis at the same site after THA is as high as 66.7% (14). Smith et al. (16) noted that IVT may dislodge intraoperatively and during other therapeutic procedures, progressing to pulmonary embolism (PE) or even death (17). Patients with LLVT have poor clinical prognostic outcomes, and post venous thrombosis syndrome can greatly distress patients and may eventually lead to amputation (9, 13). All these factors prolong the hospitalization time and increase the financial burden of patients (18). Therefore, it is important to identify patients at risk of preoperative LLVT. Preoperative LLVT in knee ligament injuries is currently understudied, and this study will help to explore this direction, identify risk factors in high-risk individuals, and develop a diagnostic tool.

This study focused on the demographic information, relevant medical history, and routine examination of patients. This study showed that the prevalence of preoperative LLVT in patients with knee ligament injuries was approximately 8.7%. Univariate logistic regression analysis showed that age, gender, comorbid cerebral infarction, damaged ligament site, injury-examination time, LDL, G, D-dimer, and FDP levels were risk factors for developing LLVT (Table 2). Among patients with preoperative LLVT, the prevalence was higher in those with advanced age, female (70.4%), combined cerebral infarction (18.5%), injury ACL/PCL+MCL/LCL (66.7%), injury-examination time ≤ 21 days (85.2%), and high levels of LDL, G, D-dimer, and FDP (Table 1). In the multivariate logistic regression analysis, gender, damaged ligament site, and D-dimer level were independent risk factors for the development of preoperative LLVT in patients with knee ligament injuries (Table 3).

Previous studies have pointed to age as a risk factor for DVT, which needs to be brought to the attention of physicians in patients of advanced age (10, 12, 13). In one study, the risk of venous thrombosis was 14.2 per 10,000 women in their 40s, increasing to 34 per 10,000 women over 45 (19). In this study, there was a significant difference between the mean age of the LLVT and None-LLVT groups, and age was shown to significantly affect the occurrence of LLVT in univariate logistic regression analysis, but not in multivariate logistic regression analysis. Considering the results of previous studies, age was included in the model construction, as increasing age causes an increase in coagulation factor levels as well as a decrease in vascular quality. Female, who have a high prevalence of knee ligament injuries in non-contact sports, also have a high prevalence of VTE (2, 20, 21). Similar results to those of previous studies were observed in this study. Some studies have shown that estrogen can promote blood clotting by increasing the levels of clotting factors II, VII, VIII, and X (22). Hormone-induced changes are a delicate balance between hemostasis and thrombosis but may increase the overall risk of thrombosis (23). In a rat study (24), injection of estradiol dipropionate (EDP) was found to increase the incidence of thrombin-induced pulmonary embolism, and the higher the estrogen level, the higher the likelihood of thrombosis.

This study showed that damaged ligament site is an independent risk factor for preoperative LLVT. Preoperative LLVT in patients with PCL injury alone was 14.3%, which was much higher than that in patients with ACL injury alone (3.7%), and patients with ACL/PCL combined with MCL/LCL injury had the highest prevalence of preoperative LLVT (15.9%). This may be related to the structure of the PCL, which is the main structure for knee extension and flexion activities and rotational activities and is twice as strong against external forces such as the ACL, which are often caused by great violence, and the injuries will lead to knee instability, pain, and weakness. This may have a greater impact on the limitation of knee motion than the ACL, and reduced lower extremity activity, among other factors, increases the risk of LLVT. D-dimer level is a significant influencing factor in thrombosis and is highly sensitive. D-dimer levels were higher in patients with proximal thrombosis than in those with distal thrombosis (25, 26).

Pharmacological anticoagulation is an important tool in the prevention of DVT, but the choice of prophylaxis is currently controversial. Related studies have suggested that early mobility exercise may be an option without routine anticoagulation prophylaxis after knee arthroscopy, but the use of low molecular heparin is recommended in patients with potential risk factors (11–13, 27). On the contrary view, aggressive preventive measures should be taken in patients with potential risk factors (10, 21, 27, 28). Wirth et al. (28) suggested that prophylaxis be given aggressively to high-risk individuals; the incidence of thrombosis receiving pharmacologic prophylaxis was 0.85%, which was much lower than the control group’s 4.1%, and the authors recommended 10 days of prophylaxis, which is effective and safe. Aggressive imaging should also be performed in high-risk patients for preoperative DVT (15). In response to this controversy, further research is needed to determine the clinical benefits of prophylactic anticoagulant use. In this study, we constructed a personalized diagnostic prediction model based on the identified risk factors, which can assist clinicians in screening patients at high risk of developing LLVT and adopting more aggressive preventive strategies for this group of patients to reduce the incidence of LLVT and alleviate the burden on patients. Our internal validation showed that the AUC of the model was 0.888 with good diagnostic efficacy, and the decision curve showed good clinical benefits.

## 5 Limitation

Our study had some limitations. Our study was a retrospective study, and there may have been some unavoidable biases. In addition, we did not find a suitable public dataset for external validation, and further validation is needed to generalize the prediction model using more data and multi-center studies. In contrast to the currently published studies, we found no significant difference in the incidence of DVT in patients who did not receive thromboprophylaxis. Therefore, prospective randomized clinical trials are needed to evaluate the clinical benefit of prophylactic anticoagulation in the perioperative period for knee ligament injuries.

## 6 Conclusion

In summary, the independent risk factors for the preoperative occurrence of LLVT in patients with knee ligament injuries were gender, damaged ligament site, and high D-dimer level. This study is the first to establish a nomogram diagnostic prediction model for the preoperative occurrence of LLVT in knee ligament injuries, which will help clinicians identify the high-risk group for preoperative LLVT and actively perform imaging screening and preventive therapeutic measures for this group.

## Data Availability

The original contributions presented in the study are included in the article/supplementary material, further inquiries can be directed to the corresponding authors.
